# A new method for assessing transverse sinus stenosis with CT venography based on the venous trans-stenotic pressure gradient

**DOI:** 10.1136/jnis-2022-019270

**Published:** 2022-10-07

**Authors:** Heyu Ding, Pengfei Zhao, Han Lv, Xiaoshuai Li, Xiaoyu Qiu, Chihang Dai, Ning Xu, Guopeng Wang, Zhenghan Yang, Shusheng Gong, Long Jin, Zhenchang Wang

**Affiliations:** 1 Department of Radiology, Beijing Friendship Hospital, Capital Medical University, Beijing, Beijing, China; 2 Department of Otolaryngology Head and Neck Surgery, Beijing Friendship Hospital, Capital Medical University, Beijing, Beijing, China; 3 Department of Intervention, Beijing Friendship Hospital, Capital Medical University, Beijing, Beijing 100050, China

**Keywords:** Catheter, CT Angiography, Intervention, Stenosis

## Abstract

**Background:**

Evaluation of the transverse sinus stenosis (TSS) is essential for TSS-related diseases.

**Objective:**

To investigate a new method for the quantitative assessment of TSS based on the correlation between TSS and trans-stenotic pressure gradient (TPG).

**Methods:**

Patients with unilateral pulsatile tinnitus with or without idiopathic intracranial hypertension were retrospectively included. All patients underwent CT venography and venous manometry and were confirmed to have TSS. The cross-sectional diameter/area of TSS, the poststenotic and prestenotic segments, and the superior sagittal sinus (SSS) were measured. The degree of TSS was calculated by dividing the diameter/area of TSS by the diameter/area of the poststenotic segment (M1/M2), prestenotic segment (M3/M4), and SSS (M5/M6). Partial correlation analysis (controlling for the effect of age, sex, outflow laterality, and contralateral stenosis) was performed to evaluate the correlation between M1–M6 and the TPG. Receiver operating characteristic curve analysis of M1–M6 for diagnosing a significant TPG (≥8 mm Hg) was performed.

**Results:**

Ninety-nine patients met the inclusion criteria. The partial correlation coefficients between M1–M6 and the TPG were 0.60, 0.61, 0.43, 0.48, 0.39, and 0.54, respectively. The areas under the curve (AUCs) of M1–M6 for diagnosing a significant TPG were 0.81, 0.86, 0.68, 0.69, 0.64, and 0.72, respectively. The AUC of M2 was significantly larger than that of M3 (P=0.002), M4 (P<0.001), M5 (P=0.001), and M6 (P<0.001).

**Conclusions:**

Quantitatively assessing TSS by taking the ratio of the cross-sectional area of TSS to that of the poststenotic segment might be a more efficient method for predicting the TPG.

**WHAT IS ALREADY KNOWN ON THIS TOPIC**

- The degree of transverse sinus stenosis (TSS) correlates with the trans-stenotic pressure gradient (TPG), but the TSS evaluation method varies.

**WHAT THIS STUDY ADDS**

- This study proposed a new method to assess the degree of TSS based on the correlation between TSS and the TPG.

**HOW THIS STUDY MIGHT AFFECT RESEARCH, PRACTICE OR POLICY**

- This new method for assessing TSS might better reflect the TPG.

## Introduction

The bilateral transverse sinus (TS) is the main intracranial venous drainage route. TS stenosis (TSS) is not uncommon in the general population, with an incidence of approximately 22%,^1^ and it has also been deeply explored for its close relationship with idiopathic intracranial hypertension (IIH), pulsatile tinnitus (PT), and chronic headache, among others.^2–6^ TSS has been suggested to be the most sensitive imaging biomarker of IIH, with 93% sensitivity and 93% specificity,^1^ and the prevalence of TSS in patients with PT is approximately 50%.^3 4^ Nevertheless, due to differences in inclusion criteria, imaging techniques, and evaluation methods, the reported incidence of TSS varies greatly within the same symptom group.

The imaging interpretation of TSS has attracted the interest of many researchers in the past two decades, but there are great differences in the imaging techniques, TSS measurement methods, and reference locations for the dural sinus. For the evaluation of the dural sinus boundary, CT venography (CTV) and contrast-enhanced magnetic resonance venography (MRV) are superior to non-contrast-enhanced MRV. For the measurement of TSS, Farb *et al* 2 adopted the diameter of the most stenotic segment based on 3D contrast-enhanced MRV, and the diameter of the superior sagittal sinus (SSS) was taken as the reference dural sinus value. In 2017, Carvalho *et al* 7 proposed the segment upstream of TSS as the reference segment. On this basis, Pellerin *et al* 8 used the ratio of the diameter of TSS to that of the upstream segment to quantitatively evaluate the degree of TSS based on the unwound TS on contrast-enhanced MRV, a method that is more objective than visual estimation. The shape of the TS is irregular; thus, the degree of TSS is poorly reflected by a single diameter. Considering this, Zhao *et al* 9 used the cross-sectional area instead of the diameter based on curved planar reformation to evaluate the degree of TSS.

The trans-stenotic pressure gradient (TPG) is an important parameter indicating the extent of cerebral venous drainage obstruction and might affect the intracranial pressure (ICP).^10 11^ The gold standard for measuring the TPG is cerebral venography with manometry, which is an invasive procedure and cannot be performed widely in clinical practice. Zhao *et al* 12 evaluated the relationship between the morphological features of TSS and the TPG on CTV, and the results showed that the degree of TSS correlated moderately with the TPG (R^2^=0.257).

We found that the sinus lumen of the segment downstream from the segment with TSS had a greater change than that of the segment upstream, which might be affected by the increased intracranial pressure, fast jet flow, or suddenly decreased venous pressure of the poststenotic segment. However, further study is needed to determine whether the TPG could be predicted more accurately based on the degree of TSS.

Therefore, we used previously reported methods and proposed a new method that takes the downstream segment as the reference to assess the degree of TSS. The correlation between the degree of TSS determined by the above methods and the TPG was further evaluated, and the purpose of this study was to identify the method that best reflected the TPG based on the degree of TSS.

## Materials and methods

### Patient population

This study was approved by the institutional review board of Beijing Friendship Hospital, Capital Medical University. A database of patients with unilateral venous PT with or without IIH, according to the diagnostic criteria established by Friedman *et al* 13 between September 2016 and July 2022, was retrospectively reviewed. All patients underwent preprocedural CTV for the presence of bilateral TSS and then diagnostic venography with venous manometry. There were no treatments or examinations for decreasing ICP, including medication use, surgery, or weight loss, during the two examinations. Spinal puncture for cerebrospinal fluid (CSF) examination would be performed in patients with clinical and imaging signs of IIH. According to the TPG, all patients meeting the inclusion criteria were divided into group A (TPG <8 mm Hg) and group B (significant TPG ≥8 mm Hg).

### CTV examination

CTV was performed using a 64-section CT scanner (Brilliance, Philips Healthcare) or a 256-section CT scanner (Revolution, GE Healthcare). The acquisition parameters were as follows: 100 kV; auto-mA; matrix, 512×512; collimation, 64×0.625 mm or 256×0.625 mm; rotation time, 0.5–0.75 s; and contrast media, 370 mg iodine/mL at 1.5 mL/kg and 5 mL/s (iopamidol, Bracco Diagnostics). The images were reconstructed with both the standard algorithm and the bone algorithm.

### Cerebral venography with venous manometry

Cerebral venography with venous manometry was performed using an angiography machine (Innova 4100-IQ, GE Healthcare, Milwaukee, Wisconsin, USA) under local anesthesia for all patients. First, conventional cerebral arteriography and venography were performed. Next, the femoral vein was accessed, and a 2.7 F microcatheter (Stride, Asahi) was navigated into the SSS from the internal carotid vein to perform direct venography on the stenotic side. A standard vascular pressure transducer (DPT-248, Yixinda) was connected to the microcatheter, and the pressure values were measured in the venous sinus segments proximal and distal to the stenotic segment on the symptomatic side. The TPG was calculated and recorded (in mm Hg).

### Data analysis

#### Evaluation of TSS on CTV

CTV images were postprocessed on a workstation (AW 4.6, GE Healthcare), and the measurements were performed using curved planar reformation images. All images were evaluated independently by two experienced neuroimaging radiologists who were blinded to the clinical and manometric data.

**Figure 1:**
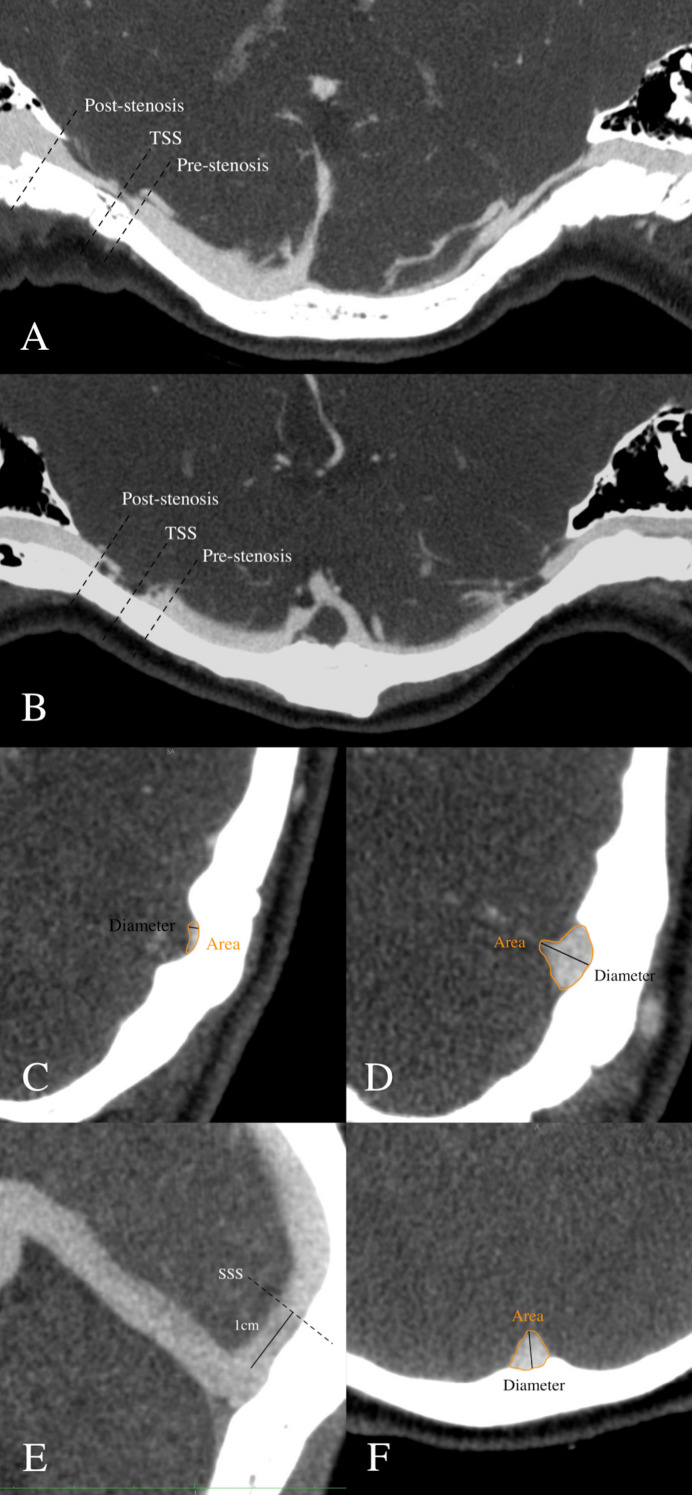
Methods for measuring transverse sinus stenosis (TSS), pre/poststenotic segments and the superior sagittal sinus (SSS). (A) Location for measuring the cross-sectional diameter/area of TSS, the prestenotic segment and the poststenotic segment for extrinsic stenosis. (B) Location for measuring the cross-sectional diameter/area of TSS, the prestenotic segment and the poststenotic segment for intrinsic stenosis. (C) Diameter and area measurement of TSS. (D) Diameter and area measurement of the pre/poststenotic segments. (E) Location for measuring the cross-sectional diameter/area of the SSS approximately one centimeter before the torcular. (F) Diameter and area measurement of the SSS.

Based on the curved planar reformation of CTV images, the cross-sectional diameter/area of the TS, TSS, and SSS on the symptomatic and asymptomatic sides was measured for each patient. Any sinus with multiple TSSs was measured at the most severe point. The same examiners measured the orthogonal diameter/area with manual electronic calipers at four locations: the focal narrowest point, the immediate poststenotic segment (downstream of the stenosis), the immediate prestenotic segment (upstream of the stenosis), and the SSS, approximately 1 cm before the torcular (figure 1). For patients with a bifid SSS,^14^ the measurement was located at 1 cm before the rostral SSS. Outflow laterality was quantitatively assessed by taking the ratio of the cross-sectional area on the asymptomatic side of the mid-TS to that on the symptomatic side of the mid-TS. All dural venous sinus measurements should avoid areas of focal stenosis, diverticula, and branching collaterals. The degree of TSS on the symptomatic and asymptomatic sides was calculated with six measurements:

M1=diameter of TSS/diameter of poststenotic segment

M2=area of TSS/area of the poststenotic segment

M3=diameter of TSS/diameter of prestenotic segment

M4=area of TSS/area of prestenotic segment

M5=diameter of TSS/diameter of SSS

M6=area of TSS/area of SSS

### Statistical analysis

All analyses were conducted using SPSS Statistics for Windows (version 26.0, IBM Corp.) and MedCalc statistical software (version 19.1, MedCalc Software). Statistical significance was defined as p<0.05 using a two-tailed hypothesis. The sample size was calculated by PASS (version 15.0, NCSS). Inter-rater reliability was determined by calculating the intraclass correlation coefficient (ICC) to avoid observer reliability bias. The coefficient was interpreted as follows: excellent (0.81–1), good (0.61–0.80), fair (0.41–0.60), and poor (0.21–0.40). Partial correlation analysis was performed to evaluate the correlation between M1–M6 and the TPG in the symptomatic side, controlling for the effect of age, sex, outflow laterality, and the degree of TSS on the asymptomatic side (M1–M6). The correlation was interpreted as follows: very strong (0.81–1.0), strong (0.61–0.80), moderate (0.41–0.60), and weak (0.21–0.40). Receiver operating characteristic (ROC) curve analysis was conducted to estimate the sensitivity and specificity of M1–M6 in predicting a significant TPG (≥8 mm Hg), and the Youden Index was used as the optimal cut-off value. ROC curve comparisons of M1–M6 for predicting a significant TPG (≥8 mm Hg) were performed using MedCalc statistical software. Further univariate linear regression analysis was performed for the method with the highest correlation coefficient and optimal diagnostic efficiency to investigate the degree to which the percentage of TSS could predict the TPG, and residuals were examined for fit.

## Results

Of ninety-nine patients (11 men, 88 women) who met the inclusion criteria, 79 cases showed a venous etiology of PT without clinically suspected IIH, and 20 cases were diagnosed with IIH accompanied by PT. The mean age was 38.7±11.7 years (range 19–75). The interval from CTV to venous manometry was 22.0 (12) days. Regarding the morphology of the sinus stenosis on the symptomatic side, 60 veins showed intrinsic stenosis (hyperplastic arachnoid villi and granulations: 58 veins: segmental hypoplasia/hyperplasia: 2 veins), 25 veins showed extrinsic stenosis (smooth tapered appearance), and 14 showed both intrinsic and extrinsic stenosis. The overall pressure gradient was 7.0 mm Hg (min–max: 1.0–24.9). A total of 55 veins were included in group A (TPG <8 mm Hg), and 44 veins were included in group B (TPG ≥8 mm Hg).

### Statistical analysis

The diameters/areas in the following locations were measured by two examiners: the focal narrowest point, the immediate poststenotic segment, the immediate prestenotic segment of the symptomatic/asymptomatic side and the SSS approximately 1 cm before the torcular or the rostral SSS. The results of variables and inter-rater reliability of variables on the symptomatic side and for the SSS are shown in table 1. The results of the partial correlation analysis controlling for the effect of age, sex, outflow laterality, and degree of TSS on the asymptomatic side between M1–M6 and the TPG are summarized in table 2. Using M2, the area of TSS divided by the area of the poststenotic segment on the symptomatic side, to interpret TSS resulted in a strong correlation with the TPG.

**Table 1:** Results of variables and inter-rater reliability for TSS, TS on the symptomatic side, and SSS between two examiners

Variables	Range (mm/mm^2^)	Mean±SD (mm/mm^2^)	ICC*
D_TSS_†	1.0–5.5	2.3±0.8	0.76
A_TSS_‡	2.3–23.0	8.7±4.9	0.84
D_post-stenosis_§	3.6–10.5	6.9±1.9	0.68
A_post-stenosis_¶	14.0–54.8	31.3±10.3	0.77
D_pre-stenosis_††	3.3–12.7	7.3±1.8	0.89
A_pre-stenosis_‡‡	13.0–62.0	34.1±12.5	0.84
D_SSS_§§	4.3–11.1	7.5±1.5	0.70
A_SSS_¶¶	17.0–54.0	34.9±8.3	0.80

*ICC, intraclass correlation coefficient.

†D_TSS_, diameter of transverse sinus stenosis on the symptomatic side.

‡A_TSS_, area of transverse sinus stenosis on the symptomatic side.

§D_post-stenosis_, diameter of poststenotic segment on the symptomatic side.

¶A_post-stenosis_, area of poststenotic segment on the symptomatic side.

††D_pre-stenosis_, diameter of prestenotic segment on the symptomatic side.

‡‡A_pre-stenosis_, area of prestenotic segment on the symptomatic side.

§§D_SSS_, diameter of superior sagittal sinus.

¶¶A_SSS_, area of superior sagittal sinus.

**Table 2:** Results of partial correlation analysis between M1–M6 and trans-stenotic pressure gradient

Method	Range	Mean±SD	R value	P value
M1	0.11–0.76	0.35±0.16	0.60	p<0.001
M2	0.02–0.83	0.28±0.19	0.61	p<0.001
M3	0.15–0.65	0.34±0.13	0.43	p<0.001
M4	0.04–0.69	0.28±0.16	0.48	p<0.001
M5	0.09–0.82	0.34±0.17	0.39	p<0.001
M6	0.02–0.80	0.26±0.17	0.54	p<0.001

**Figure 2:**
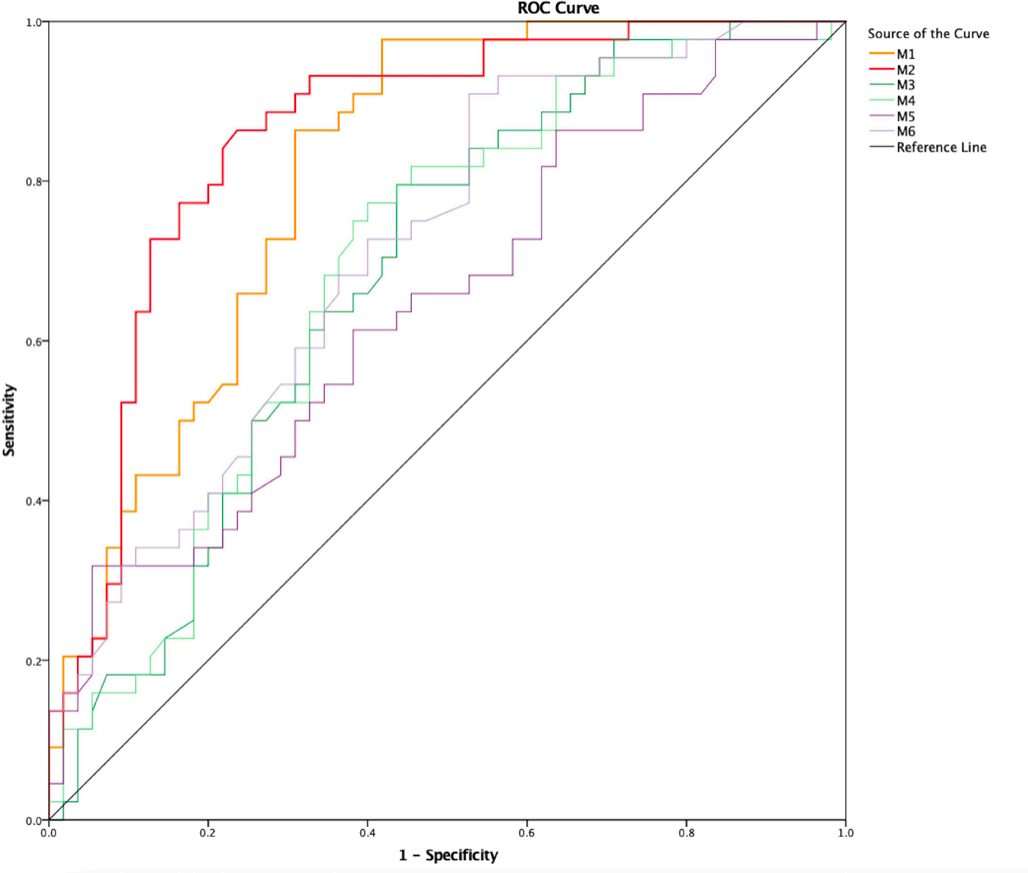
Receiver operating characteristic (ROC) curve together with specificity and sensitivity values for M1–M6 on the symptomatic side.

The area under the curve (AUC), Youden Index, cut-off point and sensitivity and specificity for the cut-off point of M1–M6 on the symptomatic side for predicting a significant pressure gradient (≥8 mm Hg) are summarized in table 3. The ROC curve together with specificity and sensitivity values for M1–M6 on the symptomatic side are shown in figure 2. On pairwise comparison of the ROC curves, the AUC of M1 was significantly higher than those of M3 (p=0.003), M4 (p=0.030), and M5 (p<0.001), and the AUC of M2 was significantly higher than those of M3 (p=0.002), M4 (p<0.001), M5 (p=0.001), and M6 (p<0.001).

**Table 3:** Area under the curve (AUC) of M1–M6 on the symptomatic side to diagnose significant trans-stenotic pressure gradient

Method	AUC (95% CI)	YI	Cut-off value	Sensitivity	Specificity
M1	0.81 (0.73 to 0.90)	0.56	0.37	0.98	0.58
M2	0.86 (0.78 to 0.93)	0.63	0.23	0.86	0.76
M3	0.68 (0.58 to 0.79)	0.36	0.34	0.80	0.56
M4	0.69 (0.59 to 0.80)	0.37	0.26	0.77	0.60
M5	0.64 (0.54 to 0.75)	0.26	0.19	0.32	0.95
M6	0.72 (0.62 to 0.82)	0.38	0.31	0.91	0.47

YI, Youden Index.

The univariate linear regression between the TPG and the degree of TSS on the symptomatic side by M2 yielded the following equation: TPG=11.13–13.39 × the degree of TSS (p<0.001). For every 10% increase in the degree of TSS by M2, the TPG increased by approximately 1.3 mm Hg.

## Discussion

We adopted different methods to assess the degree of TSS and compared their correlation with the TPG. The results showed that using the poststenotic segment as the reference dural sinus resulted in the best correlation with the TPG and showed the best diagnostic efficiency in predicting a significant TPG, while the inter-rater reliability for the area was better than the diameter of the poststenotic segment. Therefore, we recommend using the cross-sectional area of the immediate poststenotic segment as the reference location to assess the degree of TSS based on the curved planar reformation of CTV images.

The importance of assessing TSS lies in the capacity of the method to achieve a high diagnostic efficiency. Researchers agree that the point of greatest stenosis should be taken as the location of TSS, but there is still no unified standard for the reference location of the dural venous sinus. Farb *et al* 2 adopted the diameter of the distal SSS as the normal reference based on enhanced MRV, which might be because the lumen of the SSS was less variable than that of the TS, and found that it could be used to identify patients with IIH with a sensitivity and specificity of 93%. Carvalho *et al* 7 clearly defined the diameter of the immediate prestenotic segment of the TS as the reference measurement for assessing TSS, which showed better reproducibility and reduced the occurrence of false positives in diagnosing IIH.

However, there is still no conclusion as to whether the degree of TSS could be used to categorize clinical severity. Riggeal *et al* 15 calculated the percentage of TSS by dividing by the diameter of the adjacent, normal-appearing TS but found that there was no correlation between the degree of TSS and clinical severity. Qiu *et al* 16 used the cross-sectional area of TSS divided by the area of the prestenotic segment, and the results showed that there was a weak correlation between the degree of TSS and ICP. Since TSS leads to cerebral venous outflow obstruction and a further increase in the TPG, which has a close relationship with the occurrence of IIH and PT^17 18^ and is often used as the quantitative reference parameter for venous sinus stenting,^19 20^ the relationship between TSS and the TPG has been the subject of increased attention by scholars in recent years.^12 21^ In the study of West *et al*,^21^ the TPG increased by 3.5 mm Hg for every 10% increase in stenosis. In our study, for every 10% increase in the degree of TSS when taking the poststenotic segment of the TS as the reference location, the TPG increased by approximately 1.3 mm Hg, which is in accord with our previous study.^12^ Several reasons might explain the weaker effect of TSS on the TPG in our study. First, there were differences in the included subjects, and the overall TPG in our study was smaller (7 mm Hg vs 15 mm Hg). Second, the TSS interpretation methods were different.

In this study, we proposed the poststenotic segment of the TS as the reference location, and its cross-sectional area showed a strong correlation with the TPG. The CSF pressure must be higher than the dural sinus pressure to maintain CSF outflow^22^; thus, the dramatically reduced pressure in the poststenotic TS might lead to a larger CSF pressure–dural sinus pressure gradient and might have a greater influence on the sinus lumen of the poststenotic TS than that in the prestenotic TS. On the other hand, the hemodynamic changes in the poststenotic region, such as the jet flow,^23^ might play a role in the changes in the sinus wall or lumen. Third, there is variability in susceptibility to ICP elevation in different regions of the sinuses,^14^ and the poststenotic TS might be more sensitive to ICP changes than other regions. The correlation between the cross-sectional area of TSS divided by the area of the poststenotic segment and the TPG was slightly stronger than the correlation between the diameter of TSS divided by the diameter of the poststenotic segment and the TPG. The TS has a prismatic shape, with two sides that can be compressed to a limited extent at any point.^11^ Since TSS can be intrinsic, extrinsic, or both, the cross-section of the segment with TSS is always irregular, and it is difficult to take a single diameter as a measure reflecting TSS. On the other hand, the inter-rater reliability for the area of TSS (ICC=0.84) and the poststenotic segment (ICC=0.77) was better than that for the diameter (ICC=0.76 and ICC=0.68). Therefore, we recommend that the ratio of the cross-sectional area of TSS to that of the poststenotic segment should be used to measure the degree of TSS.

This study has some limitations. First, many factors correlated with the TPG apart from the degree of TSS, such as the presence of branching collaterals upstream from the stenotic segment, the length, location, or shape of the segment with TSS, and blood flow velocity/volume, which should be further comprehensively explored to establish an accurate model for predicting the TPG. Second, the enrolled population consisted of patients with PT with or without IIH, which could not fully reflect the characteristics of the TSS-related population, and the results might not be suitable for the patients with a higher TPG spectrum. On the other hand, with data from only 20 cases, there was insufficient power for comparison of the correlation between M1–M6 and the TPG separately in the IIH group. Third, contrast-enhanced CTV, which was used in this study, carries the risks of radiation, and it should be used only if it is necessary to evaluate the boundary or bone wall of the dural sinus.

## Conclusion

This study proposes a new method for assessing the degree of TSS based on the TPG. Quantitative assessment of TSS according to the ratio of the cross-sectional area of TSS to that of the poststenotic segment correlated significantly with the TPG, and this approach might be a more efficient method for reflecting the TPG via the CTV assessment of TSS. This method could be used for imaging studies of TSS-related diseases.

## Data Availability

All data relevant to the study are included in the article or uploaded as supplementary information.

## References

[1] Durst CR , Ornan DA , Reardon MA , et al . Prevalence of dural venous sinus stenosis and hypoplasia in a generalized population. J Neurointerv Surg 2016;8:1173–7. 10.1136/neurintsurg-2015-012147 26747875

[2] Farb RI , Vanek I , Scott JN , et al . Idiopathic intracranial hypertension: the prevalence and morphology of sinovenous stenosis. Neurology 2003;60:1418–24. 10.1212/01.WNL.0000066683.34093.E2 12743224

[3] Yang I-H , Pereira VM , Lenck S , et al . Endovascular treatment of debilitating tinnitus secondary to cerebral venous sinus abnormalities: a literature review and technical illustration. J Neurointerv Surg 2019;11:841–6. 10.1136/neurintsurg-2019-014725 30872352

[4] Dong C , Zhao P-F , Yang J-G , et al . Incidence of vascular anomalies and variants associated with unilateral venous pulsatile tinnitus in 242 patients based on dual-phase contrast-enhanced computed tomography. Chin Med J 2015;128:581–5. 10.4103/0366-6999.151648 25698187 PMC4834766

[5] Favoni V , Pierangeli G , Cirillo L , et al . Transverse sinus stenosis in refractory chronic headache patients: an observational study. Front Neurol 2019;10:1287. 10.3389/fneur.2019.01287 31920914 PMC6921963

[6] De Simone R , Ranieri A , Sansone M , et al . Dural sinus collapsibility, idiopathic intracranial hypertension, and the pathogenesis of chronic migraine. Neurol Sci 2019;40:59–70. 10.1007/s10072-019-03775-w 30838545

[7] Carvalho GBdaS , Matas SLdeA , Idagawa MH , et al . A new index for the assessment of transverse sinus stenosis for diagnosing idiopathic intracranial hypertension. J Neurointerv Surg 2017;9:173–7. 10.1136/neurintsurg-2016-012605 27698231

[8] Pellerin A , Aguilar Garcia J , David A , et al . A quantitative and semi-automatic measurement of transverse sinus stenosis improves idiopathic intracranial hypertension diagnostic accuracy. J Neuroradiol 2018;45:329–32. 10.1016/j.neurad.2018.05.001 29913177

[9] Zhao P , Jiang C , Lv H , et al . Why does unilateral pulsatile tinnitus occur in patients with idiopathic intracranial hypertension? Neuroradiology 2021;63:209–16. 10.1007/s00234-020-02541-6 32880675

[10] Narsinh KH , Hui F , Duvvuri M , et al . Management of vascular causes of pulsatile tinnitus. J Neurointerv Surg 2022;14:1151–7. 10.1136/neurintsurg-2021-018015 35145036 PMC9363535

[11] De Simone R , Ranieri A , Montella S , et al . The role of dural sinus stenosis in idiopathic intracranial hypertension pathogenesis: the self-limiting venous collapse feedback-loop model. Panminerva Med 2014;56:201–9. 24867405

[12] Zhao P , Ding H , Lv H , et al . CT venography correlate of transverse sinus stenosis and venous transstenotic pressure gradient in unilateral pulsatile tinnitus patients with sigmoid sinus wall anomalies. Eur Radiol 2021;31:2896–902. 10.1007/s00330-020-07415-2 33128184 PMC8043956

[13] Friedman DI , Liu GT , Digre KB . Revised diagnostic criteria for the pseudotumor cerebri syndrome in adults and children. Neurology 2013;81:1159–65. 10.1212/WNL.0b013e3182a55f17 23966248

[14] Fargen KM . A unifying theory explaining venous sinus stenosis and recurrent stenosis following venous sinus stenting in patients with idiopathic intracranial hypertension. J Neurointerv Surg 2021;13:587–92. 10.1136/neurintsurg-2020-017208 33579755

[15] Riggeal BD , Bruce BB , Saindane AM , et al . Clinical course of idiopathic intracranial hypertension with transverse sinus stenosis. Neurology 2013;80:289–95. 10.1212/WNL.0b013e31827debd6 23269597 PMC3589184

[16] Qiu X , Zhao P , Li X , et al . The relationships among transverse sinus stenosis measured by CT venography, venous trans-stenotic pressure gradient and intracranial pressure in patients with unilateral venous pulsatile tinnitus. Front Neurosci 2021;15:694731. 10.3389/fnins.2021.694731 34539330 PMC8446348

[17] Stienen A , Weinzierl M , Ludolph A , et al . Obstruction of cerebral venous sinus secondary to idiopathic intracranial hypertension. Eur J Neurol 2008;15:1416–8. 10.1111/j.1468-1331.2008.02340.x 19049565

[18] Essibayi MA , Oushy SH , Lanzino G , et al . Venous causes of pulsatile tinnitus: clinical presentation, clinical and radiographic evaluation, pathogenesis, and endovascular treatments: a literature review. Neurosurgery 2021;89:760–8. 10.1093/neuros/nyab299 34392338

[19] Fargen KM , Liu K , Garner RM , et al . Recommendations for the selection and treatment of patients with idiopathic intracranial hypertension for venous sinus stenting. J Neurointerv Surg 2018;10:1203–8. 10.1136/neurintsurg-2018-014042 30030306

[20] Abdalkader M , Nguyen TN , Norbash AM , et al . State of the art: venous causes of pulsatile tinnitus and diagnostic considerations guiding endovascular therapy. Radiology 2021;300:2–16. 10.1148/radiol.2021202584 34032509

[21] West JL , Greeneway GP , Garner RM , et al . Correlation between angiographic stenosis and physiologic venous sinus outflow obstruction in idiopathic intracranial hypertension. J Neurointerv Surg 2019;11:90–4. 10.1136/neurintsurg-2018-014004 29858399

[22] Bateman AR , Bateman GA , Barber T . The relationship between cerebral blood flow and venous sinus pressure: can hyperemia induce idiopathic intracranial hypertension? Fluids Barriers CNS 2021;18:5. 10.1186/s12987-021-00239-2 33541388 PMC7860203

[23] Ding H , Zhao P , Lv H , et al . Correlation between trans-stenotic blood flow velocity differences and the cerebral venous pressure gradient in transverse sinus stenosis: a prospective 4-dimensional flow magnetic resonance imaging study. Neurosurgery 2021;89:549–56. 10.1093/neuros/nyab222 34171923 PMC8440065

